# The Visual Movement Analysis of Physical Education Teaching considering the Generalized Hough Transform Model

**DOI:** 10.1155/2022/3675319

**Published:** 2022-03-09

**Authors:** Jianmin Liu, Yuan Li

**Affiliations:** School of Physical Education, Shandong University, Jinan 250061, China

## Abstract

In order to promote the effect of college physical education reform, this paper combines the generalized Hough transform model to analyze the visual movement of physical education teaching. The idea proposed in this paper uses the position information of the edge image itself and the direction information of the curve segment to directly eliminate the impossible targets, which fundamentally alleviates the problem of invalid sampling and accumulation. Moreover, this paper greatly constrains the parameter space based on the results of each segment of the curve, which greatly reduces the search burden of high-dimensional parameters, and combines the improved algorithm to construct a sports teaching video action analysis system. The experimental research shows that the visual movement analysis system of physical education teaching considering the generalized Hough transform model proposed in this paper can effectively analyze the sports teaching actions and improve the efficiency of physical education.

## 1. Introduction

The development of science and technology is also changing people's sports activities. As technology brings convenience to people, outdoor activities have decreased correspondingly, and people's physique has become worse and worse. Therefore, there is an urgent need to improve physical fitness through physical activities. In this context, how to cultivate students' interest in sports and how to ensure that students get the right amount of exercise every day are key considerations in school sports. Physical education in colleges and universities is an important factor in building a harmonious campus, a platform for improving the physical quality of college students, and a guarantee for improving the cohesion among students. Compared with other levels of physical education, college physical education faces a very complex environment, and physical education is affected by many factors. Only through continuous exploration in teaching practice to formulate innovative physical education models and improve the physical education system in colleges and universities can we fundamentally promote the development of college education and create a good platform for the healthy and happy growth of college students. With the continuous improvement of my country's economic level, great changes have taken place in the teaching environment of colleges and universities. Therefore, college physical education must focus on the current teaching system of colleges and universities and the characteristics of college students and formulate a diversified and innovative teaching model, so as to fundamentally optimize the current teaching structure. Changes in the teaching environment are one of the most important factors affecting teaching. Therefore, when implementing educational reforms, it is necessary to fully analyze the teaching environment of colleges and universities to implement reforms in a targeted manner to achieve the desired results.

Ordinary colleges and universities are an important carrier for the development of higher education in our country and the main position for implementing the strategy of strengthening the country with talents. The concept of core literacy should be integrated into the teaching practice of ordinary colleges and universities to improve the comprehensive quality of college students. Physical education in ordinary colleges and universities, as the main component of ordinary college curriculum teaching, is an important way to implement quality education and promote the all-round development of college students. It is urgent to implement the specific requirements of the core literacy of physical education into ordinary college physical education and cultivate the core of college students. Literacy: take into account that the realization of the physical education goals directly affects the cultivation of the core literacy of the physical education discipline. Based on the perspective of the core literacy of the physical education subject, investigate and analyze the status quo and problems in the process of achieving the goals of physical education in colleges and universities and explore and study the path to achieve these goals. Physical education activities in colleges and universities can better realize the goal of physical education in colleges and universities, which is conducive to further promoting the physical and mental health and physical fitness of college students, so that the college will become a fully developed person.

In order to promote the effect of physical education reform in colleges and universities, this paper combines the generalized Hough transform model to analyze the movement of physical education teaching videos to improve the correction effect of physical education teaching.

## 2. Related Work

The simple cell receptive field structure and calculation model of the primary visual cortex have been the focus of more than ten years of research, and many of its results have also produced a wide-ranging and far-reaching impact [1]. Literature [2] uses the Gabor function to build a model for the receptive field organization of simple cells in the primary visual cortex. Literature [3] proposed a sparse coding network model. Sparse coding is a linear model based on high-order statistical characteristics. The sparse coding model provides a more elegant calculation model for the formation mechanism of simple cell structures. Literature [4] uses the independent component analysis method to simulate the receptive field structure of simple cells. Studies have shown that the 2D receptive field profile of simple cells in the visual cortex is very similar to the core of the 2DGabor function. Both have good characteristics of local space and direction selection, and the spatial frequency and local structure of multiple directions in the local area of the image can be obtained. Features and sensitivity to the edges of the image are precisely these features, so Gabor functions are widely used in many aspects. After studying the spatial attributes of cell-based receptive field organization, people found that the complex receptive field matching calculation shows that the receptive field of cortical cells changes in time, so they must be considered as spatiotemporal entities [5]. In fact, the appearance of many simple cell receptive fields is an inseparable function of time and space, and the specific structure of the interactive elongation stimulation and inhibition zone is related to the time axis under the selectivity of these cell rates and directions. Therefore, these V1 cells are essentially spatiotemporal filters, which combine spatial and temporal information [6].

The original hierarchical model proposed in [7] believes that the output of LGN cells with overlapping receptive fields is projected to the same simple cell to form the receptive field of simple cells, the output convergent structure of simple cells with the same direction selectivity and different position selectivity. The receptive field of complex cells has been verified by biological experiments and has attracted great attention from researchers. Among them, the convergence process of simple cells and complex cells is different. The former is a linear process, and the latter is a nonlinear process. Literature [8] believes that the response of complex cells should be obtained by the MAX operation of simple cell response; that is, among simple cell populations projected to complex cells, the strongest response is the response of complex cells. Literature [9] applies machine learning methods and adopts a data-driven approach to automatically learn aggregation strategies. In recent years, researchers have discovered that there is another area outside of cell perception. Cells do not respond to visual stimuli, but only regulate the response of cells. Most of this regulation is inhibitory, which is called nonclassical receptive field inhibition or surround inhibition. [10]. Studies have shown that surround suppression can help suppress texture, enhance contours, and reduce noise and is widely used in edge detection, background and object separation, and motion information detection [11].

The human visual system accepts a large number of external visual stimuli. It does not treat these stimuli equally, but shows a certain specificity. The visual system does not respond to comprehensive stimuli, but selectively responds to some important stimuli. Respond and control: this characteristic is called the visual attention mechanism [12]. The visual attention model based on saliency proposed in [13] further embodies Koch's theoretical framework and becomes the first relatively complete computational model system in the field of attention perception research. Literature [14] believes that the attention mechanism can be divided into bottom-up attention and top-down attention. The former is data-driven and has nothing to do with high-level cognition. Literature [15] proposed a method for object recognition based on edge shape segment models. First, use the training set of calibrated object area and object center position. The learning method optimizes the selection of edge shape fragments, composes all selected edge shape fragments into a code table form, and includes the position information relative to the geometric center of the detection target in the shape fragment feature part of the code table, so that the voting method can be used to achieve target detection. When detecting the detection image, the learning method based on two-level classifier is selected to realize the detection of the target center. The process of the learning algorithm is to first extract edge shape fragments in the detected image that has a certain degree of similarity with the code table features, vote the extracted edge shape fragments according to the distance from the target center, and build a weak classifier according to this method. The recognition of the edge fragments of the object is realized by connecting the weak classifier level into a strong classifier according to the learning algorithm [16].

Literature [17] is based on the theory that the shape of the object is a unified whole and adopts the overall nature of the shape of the object. Object detection is achieved by the method of boundary structure segmentation, which has a good detection effect on translation and image deformation. Literature [18] uses a novel shape descriptor called the chord operator to describe objects.

## 3. Image Detection considering the Generalized Hough Transform Model

**Figure 1:**
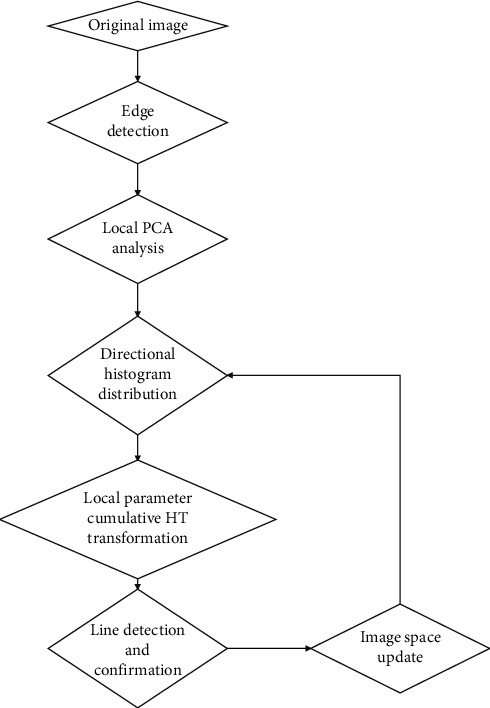
The principle of Hough straight line detection with parameter constraints based on local PCA direction analysis.

The principle of the parameter-constrained Hough line layer detection algorithm based on local PCA orientation analysis proposed in this paper is shown in Figure 1.

The algorithm includes the following key links: local PCA direction statistical analysis, local parameter space cumulative Hough transform line detection and confirmation, and image space information update. The main purpose of the statistical analysis of the local PCA direction is to obtain the statistical information of the straight line direction and create conditions for determining the local parameter HT. The cumulative Hough transform of the local parameter space greatly reduces the amount of calculation and the required storage space by reducing the search range of the parameter space. Line detection and confirmation and image spatial information update are to solve the problem of mutual influence caused by the accumulation of parallel line parameters in the classic Hough transform by deleting the detected line information.

The algorithm searches for all pixels in the edge image, and any pixel is *a*(*x*^*i*^, *y*^*i*^). We assume that *a*(*x*^*i*^, *y*^*i*^) is the center and choose a suitable square mask, the size of which is *k* × *k*, and *k* usually takes an odd number. The size of the mask directly affects the specificity and accuracy of determining the principal element direction in PCA. If the size is too large, the obtained local principal component direction is not specific enough, and if it is too small, the obtained local principal component direction will become complicated and scattered, which is not conducive to analyzing its statistical law. Experiments have proved that *k* in this idea is more appropriate to take 7, 9,11. If the number of straight lines in the image is relatively small, *k* can be selected larger, and vice versa. The set of all pixels in the mask centered on *a*(*x*^*i*^, *y*^*i*^) is called the *a*(*x*^*i*^, *y*^*i*^) support set.

PCA is a technique for analyzing data. This method can effectively find the most “main” elements and structures in the data, remove noise and redundancy, and reveal the simple structure hidden behind the complex data. This paper uses the above-mentioned characteristics of PCA to estimate the principal component direction. According to the description, by counting the pixels in the support set, the covariance matrix is obtained, and then the eigenvectors and eigenvalues are obtained. The covariance matrix is as follows [19]:

(1) $$\begin{array}{c} S=\left(\begin{array}{cc} s_{11} & s_{12}\\ s_{21} & s_{22} \end{array}\right). \end{array}$$

If the number of pixels in the support set is *n* and the coordinate of any pixel is (*x*_*i*_, *y*_*i*_), then there are

(2) $$\begin{array}{c} s_{11}=\frac{1}{n}\sum_{i=1}^{n}{\left(x_{i}-x_{m}\right)}^{2},\\ s_{12}=\frac{1}{n}\sum_{i=1}^{n}\left(x_{i}-x_{m}\right)\left(y_{i}-y_{m}\right),\\ s_{22}=\frac{1}{n}\sum_{i=1}^{n}{\left(y_{i}-y_{m}\right)}^{2}. \end{array}$$

In the formula, *x*_*m*_=1/*n*∑_*i*=1_^*n*^*x*_*i*_, *y*_*m*_=1/*n*∑_*i*=1_^*n*^*y*_*i*_ [20].

According to the description of the covariance matrix, its first characteristic root and second characteristic root are expressed as

(3) $$\begin{array}{c} \lambda_{1}=\frac{1}{2}\left\{s_{11}+s_{22}+\sqrt{{\left(s_{11}-s_{22}\right)}^{2}+4s_{12}^{2}}\right\},\\ \lambda_{2}=\frac{1}{2}\left\{s_{11}+s_{12}-\sqrt{{\left(s_{11}-s_{22}\right)}^{2}+4s_{12}^{2}}\right\}. \end{array}$$

The feature vector represents the main distribution direction of points in the support set, and the feature root represents the length of this distribution. Normally, *λ*_1_ > *λ*_2_. If the support set is an ideal straight line, then *λ*_2_=0.

The image processed by the computer is a discrete image formed after digital processes such as sampling and quantization. The straight line in the discrete space presents some characteristics that the straight line in the continuous space does not have. We summarize these characteristics and propose three guidelines that the chain code of digital straight lines should follow, as follows:

1. The 8-neighborhood chain code of a digital straight line includes at most two directions, one of which is the main direction, which is the main factor that determines the direction of the straight line.
2. The difference between the chain code values in these two directions is 1 (mod8).
3. A line segment subelement is composed of consecutive pixels with the same chain code value in the main direction, except for the first and last line segment subelement, and the lengths of the remaining line segment subelements differ by at most one pixel.

According to guidelines, the following can be understood. The straight lines in the digital image are not really straight lines. Instead, they are stepped. Therefore, the characteristic root *λ*_2_ is not necessarily equal to zero. Coupled with the influence of noise or the existence of similar straight lines, the position of edge pixels is uncertain, and *λ*_2_ is more difficult to estimate. Therefore, here only *λ*_1_ is used to find the direction *θ*′ of the supporting set principal element. If the window is an ideal straight line, *θ*′ is the inclination of the straight line in the Cartesian coordinate system.

*θ*′ is defined as follows [21]:

(4) $$\begin{array}{c} \theta^{\prime }=\;\tan^{-1}\frac{\left(\lambda_{1}-s_{11}\right)}{s_{12}}. \end{array}$$

According to the above calculation, all local direction information can be obtained. The algorithm maps it to the parameter space and then establishes the histogram corresponding to the parameter *θ* in the parameter space.

The linear polar coordinate formula is *ρ*=*x*^*∗*^*θ*′cos  *θ*+*y*^*∗*^sin  *θ*. The standard Hough transform subdivides the parameter space of (*ρ*, *θ*) during calculation and maps them to the accumulator unit.

The main purpose of this method is to constrain the polar angle range of the Hough transform to increase the detection speed and reduce the storage space. The steps are as follows:

(1) When *θ*′⟶*θ*, according to the geometric relationship, *θ*′ and *θ* have the following corresponding relationship:
  (5) $$\begin{array}{c} \theta =\left\{\begin{array}{cc} \frac{\theta^{\prime }+\pi }{2}, & \theta^{\prime }<=0,\\ \frac{\theta^{\prime }+\pi }{2}, & \theta^{\prime }>0. \end{array}\right) \end{array}$$
According to formulas (4) and (5), all possible values of *θ* are obtained.
(2) The algorithm quantizes *θ* with interval *D* and generates and draws a quantized histogram of *θ*. The threshold *t* is set reasonably according to the specific image, and the *θ*_*i*_ larger than *t* is retained. The purpose of quantization is to make the originally scattered *θ* value relatively concentrated, and the peak value is more prominent, which is easy to extract. For example, we assume *D*=5 and *t*=30. If the number of pixels satisfying *θ*=39° and *θ*=41° is greater than *t*, after quantization, only *θ*=40° can be extracted, the peak value is more obvious, and the information of *θ*=39° and *θ*=41° will not be lost, as described in step (3).
(3) According to *θ*_*i*_, the algorithm fuzzy maps the polar angle search range of the Hough transform [*θ*_*i*_ − *ε*, *θ*_*i*_+*ε*]. If the value of *ε* is too large, some unnecessary *θ* values or overlapping of subranges will be introduced. If it is too small, the effective value of *θ* will be lost. Therefore, it is proved by reasoning and experiments that $\varepsilon =\left\{\begin{array}{cc} D/2 & D\text{ Even number}\\ \left(D-1\right)/2 & D\text{ Odd number} \end{array}\right)$ and *D* is the quantization interval. This value can avoid the above two situations and at the same time avoid the impact of quantization errors. Too large a step size can lead to large errors in data loss, while a too small step size will cause peak spreading not conducive to peak extraction. Therefore, the search step sizes Δ*θ*=1 and Δ*ρ*=1 are initially selected.
(4) When the Hough transform completes the local search every time, a local fuzzy accumulation matrix is generated. The peak value is extracted from the local accumulation matrix, and the accurate parameters of the straight line are obtained. Immediately, the pixels on the extracted straight line are discarded, and the next round of search is performed. The algorithm repeats the above process until there is no straight line that meets the conditions, and the search stops. In this case, the mutual influence between the straight lines is controlled to a minimum, and the process of stratified extraction of straight lines is completed.
(5) If there are collinear points or multiple line segments in the binary image, these points and the peak voting points of the line segments in the (*ρ*, *θ*) space are extracted as the same point. Therefore, it is necessary to eliminate isolated points and small isolated line segments in the extraction result to obtain the final detection result.

For example, for a complete circle, the characteristic root of the covariance matrix obtained by edge pixel statistics has a *λ*_1_=*λ*_2_ relationship. Therefore, we assume that *λ*_1_/*λ*_2_ < *L*_*thr*_, where *L*_*thr*_ is the threshold set artificially according to the specific situation. In this way, the straight lines in the edge image and the curve segments whose curvature must not meet the circularity condition can be restricted. The algorithm initially retains the curve segment of interest, so that the calculation time is reduced.

Least Squares Analysis is a mathematical optimization technique that finds the best function match for a set of data by minimizing the sum of squared errors. Least squares method is to use the simplest method to find some absolutely unknown true values and make the sum of squared errors to be the smallest. Least Squares Fitting is usually used for curve fitting, where it is used to fit a circle.

(6) $$\begin{array}{c} A=-\frac{a}{2}, \end{array}$$

(7) $$\begin{array}{c} B=-\frac{b}{2}, \end{array}$$

(8) $$\begin{array}{c} R=\frac{\sqrt{a^{2}+b^{2}-4c}}{2}. \end{array}$$

In the formula, there are [22]

(9) $$\begin{array}{c} a=\frac{HD-EG}{CG-D^{2}},\\ b=\frac{HC-ED}{D^{2}-GC},\\ c=\frac{\sum \left(4-1\right)}{N_{i}^{2}+Y_{i}^{2}\left(\right)+a\sum X_{i}+b\sum Y_{i}},\\ C=\left(N\sum X_{i}^{2}-\sum X_{i}\sum X_{i}\right),\\ D=\left(N\sum X_{i}Y_{i}-\sum X_{i}\sum Y_{i}\right),\\ E=N\sum X_{i}^{3}+N\sum X_{i}Y_{i}^{2}-\sum \left(X_{i}^{2}+Y_{i}^{2}\right)\sum X_{i},\\ H=N\sum X_{i}^{2}Y_{i}+N\sum Y_{i}^{3}-\sum \left(X_{i}^{2}+Y_{i}^{2}\right)\sum Y_{i}. \end{array}$$

Using the method of least squares, the center and radius of the fitted circle can be derived, as shown in formulas (6), (7), and (8).

Through the above analysis, the flow of the HT circle detection algorithm constructed in this paper based on the local PCA interest parameter constraints is as follows:

1. Input the original image, the algorithm uses the edge detection operator to get the edge image, and remove the intersection point.
2. The algorithm marks the pixels in the one-value image and finds the continuous curve segment *L*_*i*_ that satisfies Length(*L*) > *L*_*thr*_, where *L*_*tir*_ is the artificially set threshold of the line segment length.
3. The algorithm performs PCA direction analysis on each segment *L*_*i*_, obtains *λ*_1_, *λ*_2_, and retains the curve segment *L*_*i*_ with *λ*_1_/*λ*_2_ < *L*_*tiv*_.
4. The algorithm fits each segment of *L*_*i*_ and retains the rough circle parameter whose fitting error is less than the set threshold.
5. The algorithm uses SHT to locally search within a limited range to obtain the circle parameters and extract the peak value.
6. The algorithm confirms the circle detection result and removes the false circle. If it is a true circle, the corresponding pixel will be deleted in the image space after the detection is completed, until all *L*_*i*_ processing is completed.

**Figure 2:**
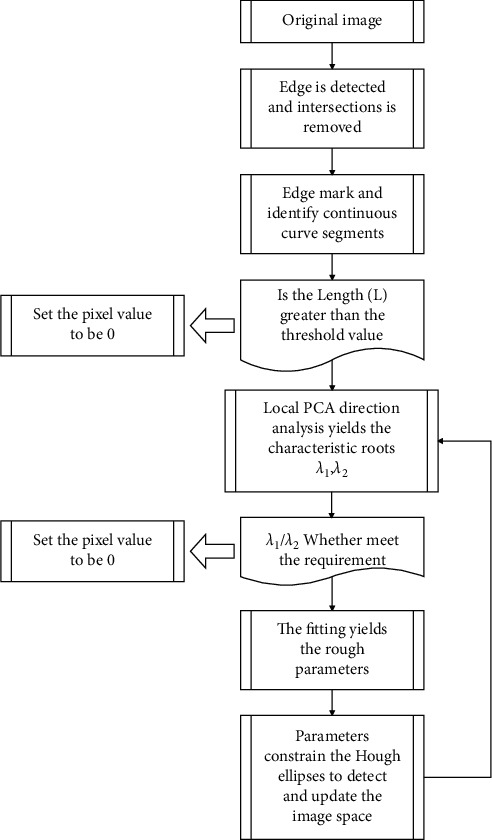
Hough multiellipse layered detection principle based on local PCA interest parameter constraints.

Figure 2 is the principle diagram of Hough multiellipse layered detection based on local PCA interest parameter constraints. The algorithm includes four key links: marking edge pixels, line segment PCA direction analysis, fitting and parameter-constrained Hough transform ellipse detection. The purpose of marking edge pixels is to separate continuous curve segments, reduce the influence between straight lines, and facilitate layered processing. Partial PCA uses the statistical analysis of each curve segment to obtain the eigenvalues of the covariance matrix to eliminate unqualified curve segments and reduce the amount of calculation. The rough ellipse parameters obtained by the fitting constrain the search range of the Hough transform, which reduces the calculation time and storage space, and improves the calculation accuracy accordingly.

For a complete elliptic curve segment, *λ*_1_ > *λ*_2_ > 0, corresponding to the major and minor axis directions of the ellipse, respectively. Therefore, by setting *λ*_1_/*λ*_2_ < *L*_*thr*_ for *λ*_1_/*λ*_2_, the line segments that do not meet the basic conditions can be preliminarily removed, and the line segments of interest can be retained.

The general representation of the quadratic curve is

(10) $$\begin{array}{c} F\left(p,X\right)=p\cdot X\\ =ax^{2}+bxy+cy^{2}+dx+ey+f=0. \end{array}$$

In the formula, $p=\left[\begin{array}{cccccc} a & b & c & d & e & f \end{array}\right],X={\left[\begin{array}{cccccc} x^{2} & xy & y^{2} & x & y & 1 \end{array}\right]}^{\text{T}}$. The polynomial *F*(*p*, *X*) is called the algebraic distance from the point (*x*, *y*) to the given quadratic curve. In order to use the least squares method to accurately fit the ellipse, many scholars impose constraints on the parameter vector *p*. In the parameter constraint method, the method of making the discriminant *b*^2^ − 4*ac* < 0 is universal and effective. However, the mandatory constraints of such inequalities are often difficult to solve. Therefore, the literature proposes that 4*ac* − *b*^2^=1, which is equivalent to *p*^*T*^  *Cp*=1, that is

(11) $$\begin{array}{c} p^{r}\left[\begin{array}{cccccc} 0 & 0 & 2 & 0 & 0 & 0\\ 0 & -1 & 0 & 0 & 0 & 0\\ 2 & 0 & 0 & 0 & 0 & 0\\ 0 & 0 & 0 & 0 & 0 & 0\\ 0 & 0 & 0 & 0 & 0 & 0\\ 0 & 0 & 0 & 0 & 0 & 0 \end{array}\right]p=1. \end{array}$$

In this way, the fitting process of the coefficients of the elliptic quadratic curve formula can be described as minimizing *E*=‖*Dp*‖^2^ is constrained by the condition *p*^*T*^*Cp*=1. $D=\left[\begin{array}{cccc} X_{1} & X_{2} & \ldots & X_{n} \end{array}\right]$ is the design matrix, and C is the restriction matrix.

When the parameters are constrained twice, the minimum distance squared problem can be solved by a nonfull rank general feature system; that is,

(12) $$\begin{array}{c} D^{\text{T}}Dp=\lambda Cp. \end{array}$$

We assume that *S*=*D*^*T*^*D*; then, there are

(13) $$\begin{array}{c} Sp=\lambda Cp. \end{array}$$

If (*λ*_*i*_, *u*_*i*_) satisfies formula (14), then (*λ*_*i*_, *μu*_*i*_) for any *μ* also satisfies the above formula. The value of *μ*_*i*_ can be obtained from *p*^*T*^*Cp*=1, as shown below:

(14) $$\begin{array}{c} \mu_{i}=\sqrt{\frac{1}{u_{i}^{T}Cu_{i}}}=\sqrt{\frac{1}{u_{i}^{T}Su_{i}}}. \end{array}$$

It can be seen from formula (14) that the feature system has six groups of eigenvalues and one eigenvector (*λ*_*i*_, *u*_*i*_), and each group can cause a local minimum. In order to ensure that the denominator in formula (14) is greater than 0, only the smallest positive feature root *λ*_*i*_ and its corresponding feature vector are required. The feature vector is the desired ellipse parameter vector, which is expressed as follows after conversion:

(15) $$\begin{array}{c} p=\left[u\text{Centre},v\text{Centre},Ru,Rv,\text{thetarad}\right]. \end{array}$$

We assume that *x*=(*x*, *y*) is the coordinate of any point on the ellipse, *u*=(*u*_1_, *u*_2_) ∈ *R*^2^ is the center of the ellipse, *ϕ* is the angle of rotation, and *a* and *b* are the semimajor and semiminor axes of the ellipse, respectively; then, the *x* point on the ellipse satisfies

(16) $$\begin{array}{c} \frac{{\left[\left(x-u_{1}\right)\cos\;\phi +\left(y-u_{2}\right)\sin\;\phi \right]}^{2}}{a^{2}}-\frac{{\left[\left(x-u_{1}\right)\sin\;\phi -\left(y-u_{2}\right)\cos\;\phi \right]}^{2}}{b^{2}}=1. \end{array}$$

The standard Hough transform detection ellipse is to map the pixels to the five-dimensional parameter space; that is, each parameter is searched globally one by one, the accumulation matrix is obtained in the parameter space, and the peak value is extracted to obtain the ellipse parameter. It can be imagined that neither SHT nor RHT calculations and accuracy are difficult to guarantee. Therefore, the article proposes the idea of using the fitting result to fuzzy constrain the search range of SHT parameters, which greatly reduces the search time while ensuring accuracy.

The fitting result is *p*=[*u*Centre, *v*Centre, *Ru*, *Rv*, thetarad], so the parameter space search range is defined as follows [23]:

1. *u*_1_ ∈ [*u*Centre − *ε*_1_, *u*Centre+*ε*_1_
2. *u*_2_ ∈ [*v*Centre − *ε*_2_, *v*Centre+*ε*_2_
3. *a* ∈ [*Ru* − *ε*_3_, *Ru*+*ε*_3_]
4. *b* ∈ [*Rv* − *ε*_4_, *Rv*+*ε*_4_]
5. *θ* ∈ [thetarad − *ε*_5_, thearad+*ε*_5_

If the value of *ε*_*i*_ ∈ [*ε*_1_,…, *ε*_5_] is too large, unnecessary calculations will be introduced, and if it is too small, useful information points will be lost. Therefore, the selection of *ε*_*i*_ should be obtained by experiment based on different images. The Hough transform performs search and voting within the constrained parameter range to obtain the final precise parameters of the ellipse.

## 4. Motion Analysis of Physical Education Teaching Videos considering the Generalized Hough Transform Model

**Figure 3:**
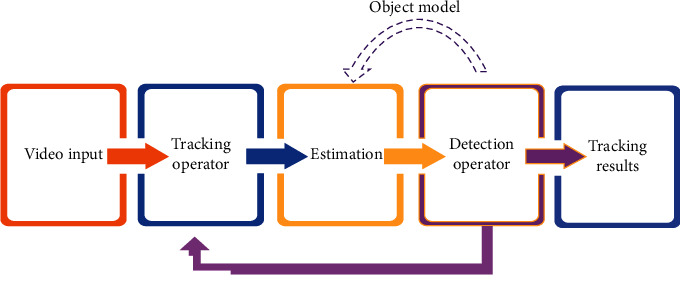
Visual movement tracking process in physical education teaching.

The proposed physical education action tracking system based on detection feedback is composed of a feedforward controller built by a tracking operator and a feedback controller built by a detection operator. The algorithm framework is shown in Figure 3. In this paper, a video sequence is used as input, and the target area to be tracked is selected and calibrated in the first frame of the video sequence.

**Figure 4:**
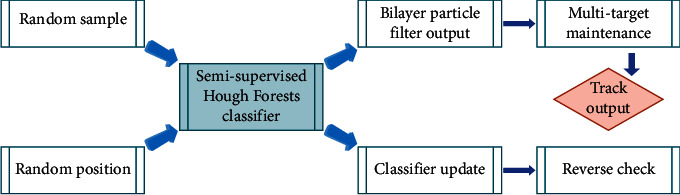
The flow of the algorithm considering the generalized Hough transform model.

Taking into account the algorithm flow of the generalized Hough transform model as shown in Figure 4, the sampling link consists of two random samples, random samples and random locations, to ensure that the features are sufficiently representative. The output of the detection operator is obtained through the semisupervised HF classifier and the two-layer particle filter framework. Then, through the goal maintenance link, the confusion of multiple goals is eliminated and the final tracking response is obtained. Finally, on the basis of the target state obtained by tracking the response, the classifier is used to redetect the results to verify the detection results, and the semisupervised HF classifier is updated with the correctly tracked samples obtained.

**Figure 5:**
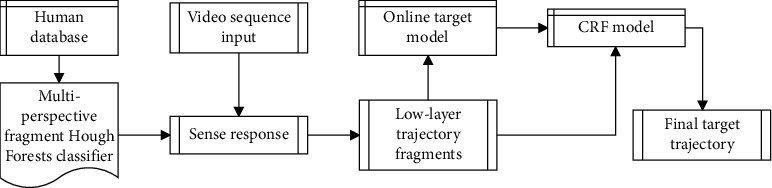
The overall framework of the algorithm.

The overall framework of the algorithm in this paper is shown in Figure 5, which is divided into two stages, offline training and online tracking. In the offline training phase, the Hough transform algorithm is used to train a general human target detection operator on the training sample set.

**Figure 6:**
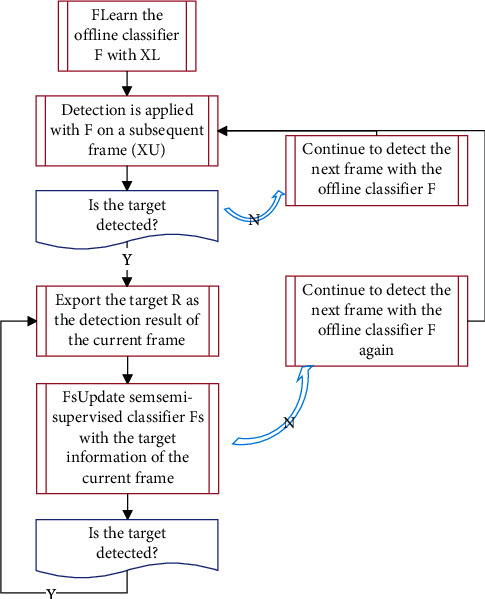
Visual movement analysis process of physical education teaching.

In the visual movement analysis of physical education, this paper adopts a random label distribution that combines the appearance of the target and space-related information to better express the apparent characteristics of the target, as shown in Figure 6.

**Figure 7:**
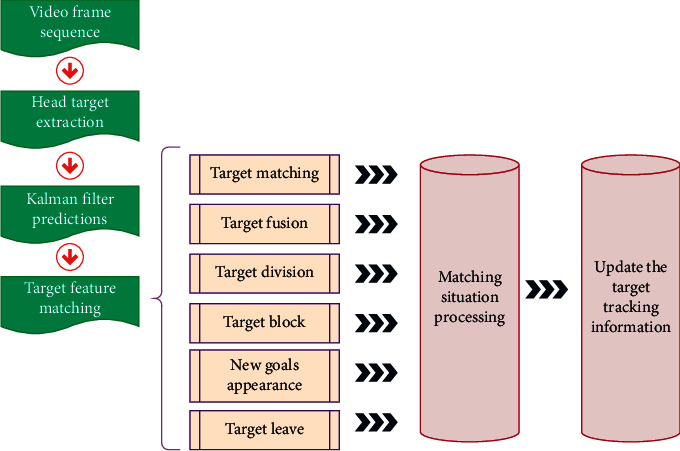
Movement tracking of physical education teaching.

In physical education, when tracking multiple targets at the same time, the Kalman tracking error will gradually increase due to the effects of occlusion and noise, so it is difficult to ensure accurate tracking for a long time, and the iterative calculation is huge. To this end, this paper proposes a tracking model as shown in Figure 7.

**Figure 8:**
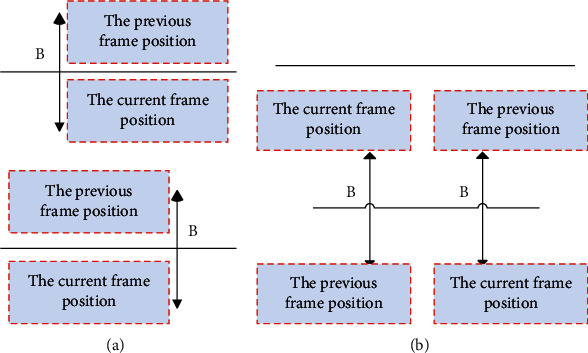
Demonstration of visual movement tracking in physical education teaching. (a) Pass through the count area. (b) Does not pass through the count area.

In order to realize the automatic statistics of the two-way flow of people at the entrance and exit, this paper delineates two counting virtual lines in the video image and regards the area between the two virtual lines as the counting area. The counting process starts only when the center point of the tracking window enters the area between the two lines. If the center point of the tracking object leaves the counting area, it means that the statistical work is over. As shown in Figure 8, Figures 8(a) and 8(b) represent two tracking targets in the PE teaching video scene.

The above constructs the visual movement analysis system of physical education teaching considering the generalized Hough transform model. On this basis, the performance of the system is analyzed. First, the tracking effect of this system in the visual analysis of physical education teaching is verified, and the results shown in Table 1 below are obtained.

From Table 1, we can see that the visual movement analysis system of physical education teaching considering the generalized Hough transform model proposed in this paper can accurately track student goals in physical education teaching. On this basis, the effect of movement feature recognition is analyzed, and the results shown in Table 2 are obtained.

From the above research, we can see that the visual movement analysis system of physical education teaching considering the generalized Hough transform model proposed in this paper can be used to accurately identify students' sports movements in physical education. After that, this paper analyzes the effect of sports visual movement, as shown in Table 3.

Through experimental research, it can be known that the visual movement analysis system of physical education teaching considering the generalized Hough transform model proposed in this paper can effectively analyze physical education movements and improve the efficiency of physical education teaching.

## 5. Conclusion

Computer vision is one of the important fields of artificial intelligence. It uses computers to complete the analysis and understanding of video images, and its technical achievements have been applied in industrial, commercial, and civil applications. Through the analysis of the students and their trajectories identified in physical education, the system can accurately count the number of students without supervision. In modern physical education where the demand for intelligent sports analysis is rapidly developing, accurate physical education movement recognition can provide effective data support for safety settings, service resource allocation, and improvement of teaching efficiency. In order to promote the effect of college physical education reform, this paper combines the generalized Hough transform model to analyze the visual movement of physical education teaching to improve the correction effect of physical education teaching. The experimental research results show that the visual movement analysis system of physical education teaching considering the generalized Hough transform model proposed in this paper can effectively analyze physical education movements and improve the efficiency of physical education teaching.

## Figures and Tables

**Table 1:** The tracking effect of the visual movement analysis system of physical education teaching considering the generalized Hough transform model.

Number	Tracking effect
1	93.28
2	96.14
3	93.73
4	94.32
5	94.60
6	93.59
7	93.58
8	95.30
9	96.90
10	94.88
11	91.45
12	94.37
13	94.26
14	96.18
15	95.69
16	96.66
17	96.32
18	96.62
19	96.27
20	96.26
21	91.83
22	92.59
23	91.74
24	96.04
25	93.49
26	94.54
27	92.88
28	92.22
29	96.20
30	95.79
31	92.59
32	96.30
33	95.07
34	95.73
35	92.04
36	92.51
37	91.71
38	94.46
39	91.65
40	93.26
41	93.21
42	91.23

**Table 2:** The movement recognition effect of the visual movement analysis system of physical education teaching considering the generalized Hough transform model.

Number	Action recognition
1	88.30
2	90.53
3	92.62
4	92.20
5	92.08
6	87.10
7	87.25
8	91.94
9	90.41
10	87.95
11	88.05
12	91.57
13	90.05
14	87.69
15	89.70
16	90.46
17	87.96
18	92.02
19	92.81
20	87.42
21	92.46
22	89.34
23	92.14
24	87.90
25	91.03
26	92.87
27	87.62
28	87.84
29	92.34
30	90.16
31	88.97
32	90.02
33	87.56
34	92.57
35	88.98
36	88.04
37	91.99
38	90.82
39	92.50
40	87.83
41	88.94
42	91.27

**Table 3:** The movement analysis effect of the visual movement analysis system of physical education teaching considering the generalized Hough transform model.

Number	Action analysis
1	87.61
2	87.28
3	84.72
4	82.63
5	84.00
6	87.05
7	90.48
8	83.22
9	82.46
10	82.76
11	85.09
12	84.74
13	85.56
14	87.70
15	86.60
16	83.16
17	83.31
18	90.46
19	85.20
20	84.40
21	84.25
22	85.35
23	84.52
24	88.50
25	85.37
26	83.42
27	88.05
28	85.33
29	87.14
30	89.87
31	89.10
32	82.73
33	87.27
34	84.70
35	87.12
36	82.79
37	89.01
38	90.35
39	83.40
40	85.19
41	85.91
42	86.66

## Data Availability

The labeled dataset used to support the findings of this study are available from the corresponding author upon request.

## References

[1] Gu R., Wang G., Jiang Z., Hwang J. N. (2019). Multi-person hierarchical 3d pose estimation in natural videos. *IEEE Transactions on Circuits and Systems for Video Technology*.

[2] Nasr M., Ayman H., Ebrahim N., Osama R., Mosaad N., Mounir A. (2020). Realtime multi-person 2D pose estimation. *International Journal of Advanced Networking and Applications*.

[3] Thành N. T., Công P. T. (2019). An evaluation of pose estimation in video of traditional martial arts presentation. *Journal of Research and Development on Information and Communication Technology*.

[4] Petrov I., Shakhuro V., Konushin A. (2018). Deep probabilistic human pose estimation. *IET Computer Vision*.

[5] Hua G., Li L., Liu S. (2020). Multipath affinage stacked—hourglass networks for human pose estimation. *Frontiers of Computer Science*.

[6] Aso K., Hwang D. H., Koike H. Portable 3D human pose estimation for human-human interaction using a chest-mounted fisheye camera.

[7] Mehta D., Sridhar S., Sotnychenko O. (2017). VNect. *ACM Transactions on Graphics*.

[8] Liu S., Li Y., Hua G. (2018). Human pose estimation in video via structured space learning and halfway temporal evaluation. *IEEE Transactions on Circuits and Systems for Video Technology*.

[9] Ershadi-Nasab S., Noury E., Kasaei S., Sanaei E. (2018). Multiple human 3d pose estimation from multiview images. *Multimedia Tools and Applications*.

[10] Nie X., Feng J., Xing J., Xiao S., Yan S. (2018). Hierarchical contextual refinement networks for human pose estimation. *IEEE Transactions on Image Processing*.

[11] Nie Y., Lee J., Yoon S., Park D. S. (2019). A multi-stage convolution machine with scaling and dilation for human pose estimation. *KSII Transactions on Internet and Information Systems (TIIS)*.

[12] Zarkeshev A., Csiszár C. (2019). Rescue method based on V2X communication and human pose estimation. *Periodica Polytechnica: Civil Engineering*.

[13] McNally W., Wong A., McPhee J. (2018). Action recognition using deep convolutional neural networks and compressed spatio-temporal pose encodings. *Journal of Computational Vision and Imaging Systems*.

[14] Díaz R. G., Laamarti F., El Saddik A. (2021). DTCoach: your digital twin coach on the edge during COVID-19 and beyond. *IEEE Instrumentation and Measurement Magazine*.

[15] Bakshi A., Sheikh D., Ansari Y., Sharma C., Naik H. (2021). Pose estimate based yoga instructor. *International Journal of Recent Advances in Multidisciplinary Topics*.

[16] Colyer S. L., Evans M., Cosker D. P., Salo A. I. T. (2018). A review of the evolution of vision-based motion analysis and the integration of advanced computer vision methods towards developing a markerless system. *Sports medicine - open*.

[17] Sárándi I., Linder T., Arras K. O., Leibe B. (2020). Metrabs: metric-scale truncation-robust heatmaps for absolute 3d human pose estimation. *IEEE Transactions on Biometrics, Behavior, and Identity Science*.

[18] Azhand A., Rabe S., Müller S., Sattler I., Heimann-Steinert A. (2021). Algorithm based on one monocular video delivers highly valid and reliable gait parameters. *Scientific Reports*.

[19] Xu J., Tasaka K. (2020). [Papers] keep your eye on the ball: detection of kicking motions in multi-view 4K soccer videos. *ITE Transactions on Media Technology and Applications*.

[20] Li Z., Bao J., Liu T., Jiacheng W. (2020). Judging the normativity of PAF based on TFN and NAN. *Journal of Shanghai Jiaotong University*.

[21] Bhombe J., Jethwa A., Singh A., Nagarhalli T. (2021). Review of pose recognition systems. *VIVA-Tech International Journal for Research and Innovation*.

[22] Nagalakshmi Vallabhaneni D. P. P. (2021). The analysis of the impact of yoga on healthcare and conventional strategies for human pose recognition. *Turkish Journal of Computer and Mathematics Education (TURCOMAT)*.

[23] Liu J.-J., Newman J., Lee D.-J. (2021). Using artificial intelligence to provide visual feedback for golf swing training. *Electronic Imaging*.

